# Establishment of national standard for anti-SARS-Cov-2 neutralizing antibody in China: The first National Standard calibration traceability to the WHO International Standard

**DOI:** 10.3389/fimmu.2023.1107639

**Published:** 2023-02-14

**Authors:** Lidong Guan, Qunying Mao, Dejiang Tan, Jianyang Liu, Xuanxuan Zhang, Lu Li, Mingchen Liu, Zhongfang Wang, Feiran Cheng, Bopei Cui, Qian He, Qingzhou Wang, Fan Gao, Yiping Wang, Lianlian Bian, Xing Wu, Jifeng Hou, Zhenglun Liang, Miao Xu

**Affiliations:** ^1^ Institute of Biological Products, National Institutes for Food and Drug Control, Beijing, China; ^2^ National Health Commission Key Laboratory of Research on Quality and Standardization of Biotech Products, National Institutes for Food and Drug Control, Beijing, China; ^3^ National Medical Products Administration Key Laboratory for Quality Research and Evaluation of Biological Products, Institute of Biological Products, National Institutes for Food and Drug Control, Beijing, China; ^4^ Guangzhou Laboratory, Guangzhou, China

**Keywords:** international standard (IS), national standard (NS), COVID-19, neutralizing antibody (NtAb), traceability, live virus neutralization assay (Neut), pseudovirus neutralization assay (PsN)

## Abstract

Neutralizing antibody (NtAb) levels are key indicators in the development and evaluation of severe acute respiratory syndrome coronavirus-2 (SARS-CoV-2) vaccines. Establishing a unified and reliable WHO International Standard (IS) for NtAb is crucial for the calibration and harmonization of NtAb detection assays. National and other WHO secondary standards are key links in the transfer of IS to working standards but are often overlooked. The Chinese National Standard (NS) and WHO IS were developed by China and WHO in September and December 2020, respectively, the application of which prompted and coordinated sero-detection of vaccine and therapy globally. Currently, a second-generation Chinese NS is urgently required owing to the depletion of stocks and need for calibration to the WHO IS. The Chinese National Institutes for Food and Drug Control (NIFDC) developed two candidate NSs (samples 33 and 66–99) traced to the IS according to the WHO manual for the establishment of national secondary standards through a collaborative study of nine experienced labs. Either NS candidate can reduce the systematic error among different laboratories and the difference between the live virus neutralization (Neut) and pseudovirus neutralization (PsN) methods, ensuring the accuracy and comparability of NtAb test results among multiple labs and methods, especially for samples 66–99. At present, samples 66–99 have been approved as the second-generation NS, which is the first NS calibrated tracing to the IS with 580 (460–740) International Units (IU)/mL and 580 (520–640) IU/mL by Neut and PsN, respectively. The use of standards improves the reliability and comparability of NtAb detection, ensuring the continuity of the use of the IS unitage, which effectively promotes the development and application of SARS-CoV-2 vaccines in China.

## 1 Introduction

The coronavirus disease 2019 (COVID-19) outbreak has caused over 620 million confirmed infections worldwide and at least 6.5 million deaths (1). To effectively prevent and control the COVID-19 epidemic, the research and development of the COVID-19 vaccine was rapidly conducted at an unprecedented global scale and investment. Currently, 198 vaccines are in the preclinical stage, 169 have entered clinical trials, and 43 have been approved for market application or emergency use (2, 3). Over 12 billion vaccine doses have been administered worldwide (1). Thirteen vaccines have been approved for market application or emergency use in China, and over 3.4 billion vaccine doses have been administered (3, 4). However, owing to the continuous emergence of variants of concern (VOC) and their immune escape ability, vaccine research and development has shifted from the original prototype strain vaccine to the multi-conjugate, multivalent, broad-spectrum, and pan-coronavirus vaccines with prototype strains as one of the components (5–11).

The level of neutralizing antibodies (NtAb) is an important indicator of vaccine effectiveness (12–14) and a key indicator in the study of treatment and population seroepidemiology. The accuracy, comparability, and reliability of the test results are of great significance for vaccine development, production, and application (15). At present, the commonly used detection methods mainly include the live virus neutralization (Neut) and pseudovirus neutralization (PsN) methods (16–18). Between them, the traditional Neut method is the internationally recognized gold standard, but it requires the use of live virus and live cells, which needs to be carried out in a level three biosafety laboratory, and is greatly affected by many influencing factors, such as the detection of virus strains, cells, other living matrices, and personal subjective judgment (19). Although the PsN method has a short detection cycle, high biological safety, and objective detection results, it also requires using live cells and artificially constructed pseudovirus (19). Importantly, it is difficult to compare the effectiveness of vaccines in different clinical trials due to the use of different testing methods and laboratories, which has become a key challenge in the management of the COVID-19 pandemic for the World Health Organization (WHO) and regulatory agencies worldwide. Especially in the case of the global development of COVID-19 vaccines with the use of multi-technology routes, involvement of multiple centers, rapid synchronous development, and clinical evaluation (20), the timely establishment of accurate and reliable NtAb standards for COVID-19 vaccines is of great importance.

Therefore, China’s NIFDC and WHO invited experts to establish the first National Standard (NS) for the COVID-19 neutralizing antibody (No. 280034-202001) and the first WHO International Standard (IS) for anti-SARS-CoV-2 immunoglobulin (Coded: 20/136) in September and December 2020, respectively (21, 22). The NtAb potencies were 1000 units/mL and 1000 International Units (IU)/mL, respectively. These two standards were expected to guarantee and promote the effective evaluation of COVID-19 vaccines, which was known to be the principal role of the WHO and Chinese national regulations in the prevention and control of the COVID-19 epidemic. At present, the inventory of the first NS in China is about to be depleted. To meet the needs of vaccine research, development, and application in China, and trace the Chinese national standards to IU and ensure the unity of IU worldwide, the research and development of the new generation of standards is imminent. In 2021, the Chinese National Institutes for Food and Drug Control (NIFDC) in conjunction with other companies and institutes prepared two candidates using the convalescent plasma and immunoglobulin of patients with COVID-19 collected before April 2021, respectively. After homogeneity and stability research, nine relevant laboratories in China were invited to conduct a collaborative calibration study, and a NS with calibrated tracing to IS was established. This study describes the results of cooperative calibration and traceability of the Chinese NS to the IS, to provide uniform and coordinated global standards for the promotion of vaccine research, development, production, and application in China.

## 2 Materials and methods

### 2.1 Materials and ethics statement

Thirteen plasma samples from COVID-19 convalescent patients collected before April 2021 and one batch anti-SARS-CoV-2 immunoglobulin (Lot: 20200905) were generously provided by Sinopharm Wuhan Plasma derived Biotherapies Co., Ltd. All donors gave informed consent for the use of their plasma.

### 2.2 Production and tests of the candidates

Candidate 1 (Lot:202102) was a frozen preparation of a pool of plasma from 13 individuals infected with one of the early 2021 SARS-CoV-2 isolates. After heat-inactivation for 30 min at 56°C and defibrination, the pooled plasma was aseptically aliquoted in glass DIN ampoules, each containing 0.2 mL, which were sealed and cryopreserved at –35°C. The Candidate 1 preparation was also tested for markers of known blood-borne virus infections (HBsAg, HIV-1/HIV-2 antibody, HCV antibody, and syphilis antibody) and was found to be negative.

Candidate 2 is a freeze-dried preparation of anti-SARS-CoV-2 immunoglobulin, which was prepared by the company according to the immunoglobulin manufacturing process (Lot: 20200905). It was aseptically aliquoted in glass DIN ampoules, each containing 0.5 mL, and lyophilized, sealed, and cryopreserved at –35°C.

The absolute NtAb titers of candidates 1 and 2 were 1650 and 566 detected by the PsN method, respectively, with good homogeneity (geometric coefficient of variation [GCV] between samples: 24% and 33%, respectively). The stabilities of the two candidates were assessed using an accelerated thermal degradation study. The ampoules of the two candidates were stored at different temperatures: –35 (baseline), +4, +20, and +37°C for two weeks and one month. The potencies relative to the –35°C baseline were calculated. Real-time data on the degradation of candidate 1 and 2 samples showed no loss of potency for up to a month and performed well in terms of stability (**Supplementary Figure 1**).

### 2.3 Collaborative calibration study

#### 2.3.1 Samples and virus

The collaborative study sample consisted of 10 samples, as summarized in **Supplementary Table 1** (coded 10, 11, 22, 33, 44, 55, 66, 77, 88 and 99, respectively). Sample 11 was the first WHO International Standard for anti-SARS-CoV-2 immunoglobulin (coded 20/136), purchased from NIBSC (20). Sample 22 was the first national standard of China for anti-SARS-CoV-2 immunoglobulin (Lot 280034-202001) and was stored in our unit (19). Sample 99 was a duplicate of sample 66 (candidate 1). Sample 33 (candidate 2; Lot: 20200905) was provided by NIFDC. Samples 10 and 44 were SARS-CoV-2-negative healthy human serum. Samples 55 and 77 were convalescent sera from two donors infected with SARS-CoV-2 provided by the Boya Biopharmaceutical Group Co., Ltd., with lower or higher titers, respectively. Sample 88 was a pool of sera from COVID-19 recovered patients collected in Guangzhou Laboratory with sequence-confirmed infection with the Delta variant.

The methods used by the participants were in-house Neut, and PsN assays. The PsN assay used a non-replicative vesicular stomatitis virus (VSV)-based pseudotype virus provided by the NIFDC and commercial company.

#### 2.3.2 Participants

Nine laboratories with NtAb detection experience agreed to participate in the study, including Jiangsu Provincial Center for Disease Control And Prevention, Sinovac Life Science Co., Ltd., Guangzhou Laboratory, Institute of Medical Biology, Chinese Academy of Medical Sciences, Wuhan Institute of Biological Products Co., Ltd., Institute of Biotechnology, Academy of Military Medical Sciences, Beijing Institute of Biological Products Co., Ltd., the Division of HIV/acquired immunodeficiency syndrome (AIDS) and Sexually Transmitted Virus Vaccines, and the Division of Blood Products, NIFDC. All laboratories were referred to by a code number from one to nine, randomly allocated.

#### 2.3.3 Collaborative calibration study

The NIFDC organized this collaborative study. Participants were requested to test the study samples using their established methods, including Neut and PsN assays, for the detection of antibodies against the wild-type (WT) SARS-CoV-2 and Delta variant.

The Neut assay, for the detection of anti-SARS-CoV-2 antibodies, is a cytopathic effect-based microneutralization assay (23). The PsN method was performed according to Nie et al. (24). The participants were asked to perform three independent assays for each challenged strain on different days. At least eight dilutions were suggested for each assay for each sample, and at least four wells were set for each dilution in parallel.

### 2.4 Statistical methods

The raw data were submitted to the NIFDC. The end-point titer of each sample was calculated from the 50% inhibitory dilutions (ID_50_) provided by the participants using the NIFDC biostats software. To be calibrated by the WHO IS, the relative potency of each sample against the WHO IS was calculated by taking the end- point titer ratio of sample/WHO IS in same assay and multiplying assigned value of the first WHO IS (1000 IU/mL). All log-transformed data were analyzed using a probit model. Model fit was assessed using analysis of variance. Variabilities between laboratories and assays are expressed using GCV. The calculation and analysis software used included Microsoft Excel 2016 (Microsoft corporation, USA, 2016), NIFDC Biostat 1.0 (NIFDC, China,2019), and JMP 13(SAS Institute, Cary, NC, 1989-2016).

## 3 Results

### 3.1 Collaborative calibration and feedback data

Nine Chinese labs with experience in testing SARS-CoV-2 NtAb, including two national vaccine quality control laboratories, one national laboratory, one disease prevention and control agency, four vaccine manufacturers, and one research institute participated in this collaborative calibration study. All labs returned results according to requirements. The Neut method was adopted by six laboratories, and the PsN method was adopted by four laboratories. In each method, three independent and effective tests were performed on all samples using the WT and Delta strains, respectively.

**Figure 1:**
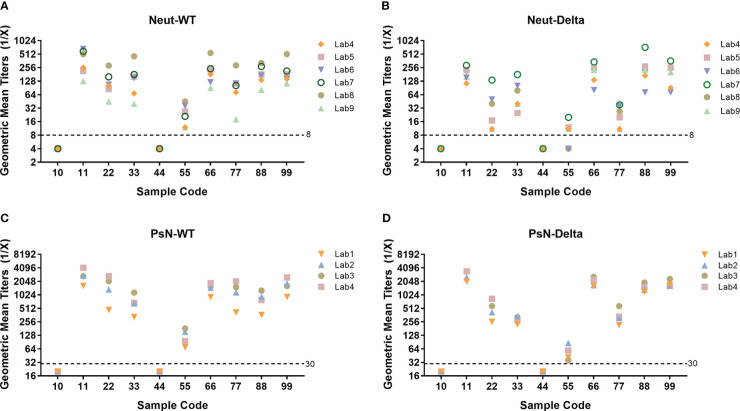
Neutralizing antibody (NtAb) geometric mean titers (GMT) of all collaborative calibration study samples across all participants. **(A)** Live virus neutralization assay (Neut), WT strain; **(B)** Neut assay, Delta variant; **(C)** Pseudovirus neutralization assay (PsN), WT strain; **(D)** PsN assay, Delta variant. Nine laboratories with experience in testing SARS-CoV-2 NtAbs participated in this collaborative calibration study. The Neut method was adopted by six laboratories, and the PsN method was adopted by four laboratories. In each laboratory, three independent and effective tests were performed on all samples using the WT and Delta strains, respectively. All laboratories returned results according to requirements.

The geometric mean titer (GMT) results for all samples from the nine laboratories are presented in **Figure 1**. The assay results for all laboratories showed the same trend. All the results of the negative samples (Nos. 10 and 44) were negative, and the coincidence rate was 100%. Among the eight positive samples, negative results for samples 55 and 77 were found among some Neut-Delta detection assays from three laboratories. The 2/3, 3/3, and 3/3 negative results for sample 55 were obtained in Lab4, Lab8, and Lab6, respectively. The 2/3 negative results for sample 77 were obtained in Lab4. The remaining six samples tested positive. Statistical analysis showed that all the data met the validity of the model. In total, 480 results using the Neut method (WT, Delta) and PsN method (WT, Delta) were analyzed using the Grubbs test. All the results had no outliers at the 5% significance level; therefore, all data were included in the subsequent analyses.

### 3.2 Intra-assay and inter-assay variability

The relative potencies of the coded duplicate samples of candidate standard 1 (samples 66 and 99) were used to assess intra-assay variability and are shown in **Supplementary Figure 2**. For the Neut assay, relative potencies ranged between 0.5 and 2.0 in 93% of cases challenged with both the WT and Delta strains, with the exception of one result in Lab5 and Lab9 (each challenged with the WT strain) and one result in Lab4 and Lab5 (each challenged with the Delta strain). For PsN, the relative potencies ranged between 0.5 and 2.0 in 100% of cases challenged with both the WT and Delta strains. The results showed a good level of intra-assay precision among the participating laboratories, with better precision in the PsN method (**Supplementary Figure 2**).

Inter-assay variability, as illustrated by the between-assay GCVs in **Supplementary Table 2**, ranged from 0% to 105.7% (4.0-fold difference in the results obtained for sample 33 by Lab6) for the Neut assay challenged with the WT strain, and ranged from 0% to 163.3% (5.3-fold difference in the results obtained for sample 33 by Lab8) for the Neut assay challenged with the Delta strain. For the PsN assay, the inter-assay variability ranged from 3.2% (1.1-fold difference in the results obtained for sample 66 by Lab3) to 78.6% (3.1-fold difference in the results obtained for sample 55 by Lab3) when challenged by WT, and from 5.3% (1.1-fold difference in the results obtained for sample 66 by Lab1) to 79.5% (3.1-fold difference in the results obtained for sample 33 by Lab3) when challenged with the Delta strain, respectively. Good inter-assay variability was found for both the Neut and PsN assays among the participating laboratories. Better intra-assay variability was also found in the PsN method compared with that in the Neut method, consistent with the performance of these methods.

### 3.3 Collaborative calibration results

**Figure 2:**
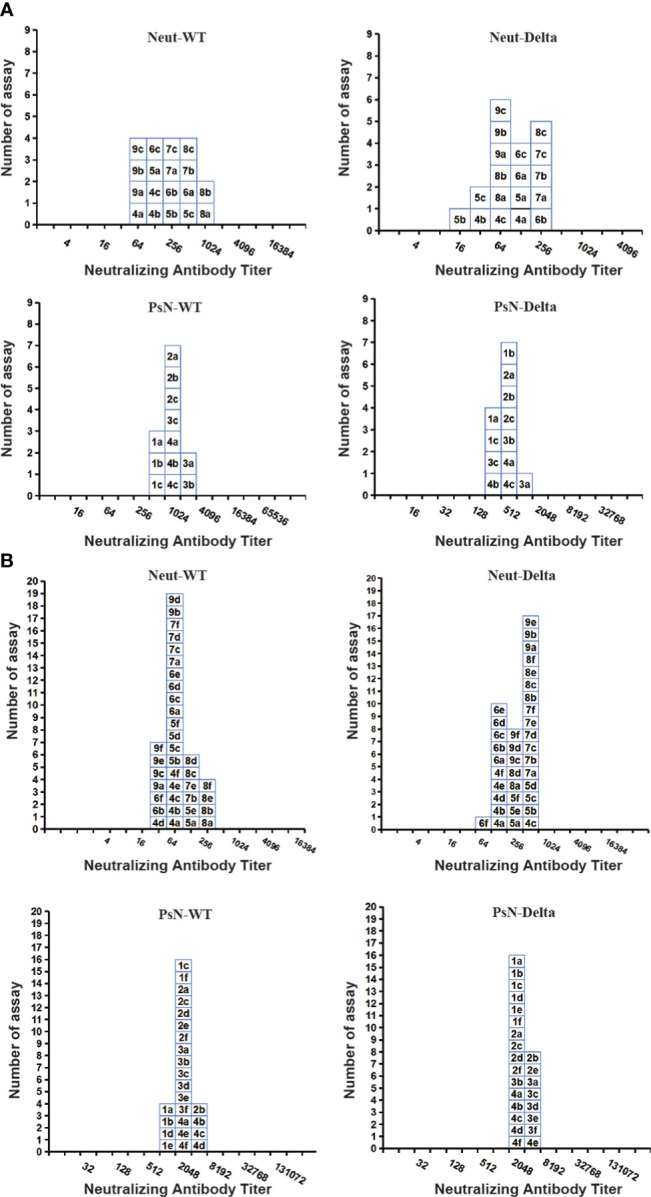
Histograms showing the distribution of the endpoint titer (ID_50_) for two candidate national standards (samples 33 and 66–99) across laboratories. **(A)** Sample 33, **(B)** Samples 66–99. Each box represents the endpoint titer for each assay, labelled with the laboratory code number (1–9), and followed by one independent assay (a–f), indicating the assays. The live virus neutralization assay (Neut) and pseudovirus neutralization assay (PsN) results using the wild-type (WT) strain or Delta variant are shown side by side.

The test results of each laboratory of the two candidate standards (samples 33 and 66–99) were subjected to statistical and frequency distribution analyses. As shown in **Figure 2**, when challenged with the WT strain, the GMTs of the Neut method for samples 33 and 66–99 were 133 (86–205) and 194 (157–238), respectively. The GMT of the PsN method were 641 (468–878) and 1512 (1287–1776), respectively. The frequency distribution shows that the peak pattern of samples 66–99 is more symmetrical and sharper than that of sample 33. Statistically, both were normally distributed (P>0.05).

When challenged with the Delta strain, the GMTs of the Neut method for samples 33 and 66–99 were 62 (41–94) and 186 (152–227), respectively, whereas those of the PsN method were 289 (227–368) and 1889 (1695–2106), respectively. Statistically, the results of samples 33 and 66–99 were also normally distributed (P>0.05), but the result distribution peak of the Neut method challenged with the Delta strain was not as symmetric and sharp as that of the WT strain.

#### 3.3.1 Inter-laboratory variability

To assess the effect of test results calibrated by samples 33 and 66–99 from different laboratories, the neutralization relative titers (RT) for each sample were expressed relative to samples 33 and 66–99 in each assay, respectively.

For the neutralizing results detected using the WT strain, the GCV of the endpoint titer for each sample between all participants were 64–148% and 36–115% in the Neut and PsN methods, respectively (**Table 1**). Relative to sample 33, the GCV of RT/33 among all participants decreased to 34–94% and 12–55% using the Neut and PsN methods, respectively. Relative to samples 66–99, the GCV of RT/66–99 among all participants decreased to 29–88% and 7–54%, respectively. Samples 33 and 66–99 effectively reduced the variability among different laboratories. Samples 66–99 were more effective than sample 33 in reducing the inter-laboratory GCV and could reduce the difference in the detection of all samples (including Delta convalescent serum, sample 88).

**Table 1:** Geometric Coefficients of Variation (%, GCV) of endpoint titer and relative titers against sample 33 or samples 66–99 across all participants.

Challenge Virus	Assay type	%, GCV	Sample Code							
			*11*	*22*	*33*	*55*	*66*	*77*	*88*	*99*
WT	Neut	Endpoint	93	86	132	74	86	148	64	70
		RT/33	94	47	/	48	55	34	49	54
		RT/66–99	88	29	55	53	/	80	31	/
	PsN	Endpoint	46	115	67	56	36	100	70	52
		RT/33	51	55	/	27	39	49	12	51
		RT/66–99	7	54	39	51	/	42	43	/
Delta	Neut	Endpoint	40	167	107	33	69	73	111	90
		RT/33	95	57	/	88	142	71	149	148
		RT/66–99	43	175	143	50	/	110	38	/
	PsN	Endpoint	28	65	20	48	25	50	23	18
		RT/33	23	55	/	43	24	35	19	22
		RT/66–99	18	54	24	69	/	27	5	/

Green represents lower GCV of relative titers than that of endpoint titers, and red represents higher GCV of relative titers than that of endpoint titers.

To assess the suitability of samples 33 and 66–99, the neutralization relative titers (RT) for each sample were calculated relative to samples 33 and 66–99 in each assay. The GCV of the endpoint titer, RT/33, and RT/66–99 among all participants was statistically analyzed to reflect variability between different laboratories. WT, wild-type; PsN, pseudovirus neutralization assay; Neut, live virus neutralization assay.

For the neutralizing results detected using the Delta variant, the GCV of the endpoint titer among all participants were 33–167% and 18–65% in the Neut and PsN methods, respectively (**Table 1**). Relative to sample 33, the GCV of RT/33 for all participants decreased to 57–149% and 19–55% for the Neut and PsN methods, respectively. Relative to sample 66–99, the GCV of RT/66–99 for all participants decreased to 38–175% and 5–69%, respectively. It is suggested that when challenged with the Delta variant, the use of samples 33 and 66–99 can only reduce the detection difference of some samples and even lead to an increase in the inter-laboratory difference of most samples. The results indicate that the two candidates are not suitable for the Neut and PsN methods using the Delta variant.

#### 3.3.2 Inter-method variability

To assess the effects of candidates 33 and 66–99 on different detection methods, the correlation between the two methods before and after calibration, and the GMT ratio (GMT Neut method/GMT PsN method) were analyzed.

##### 3.3.2.1 Correlation

**Figure 3:**
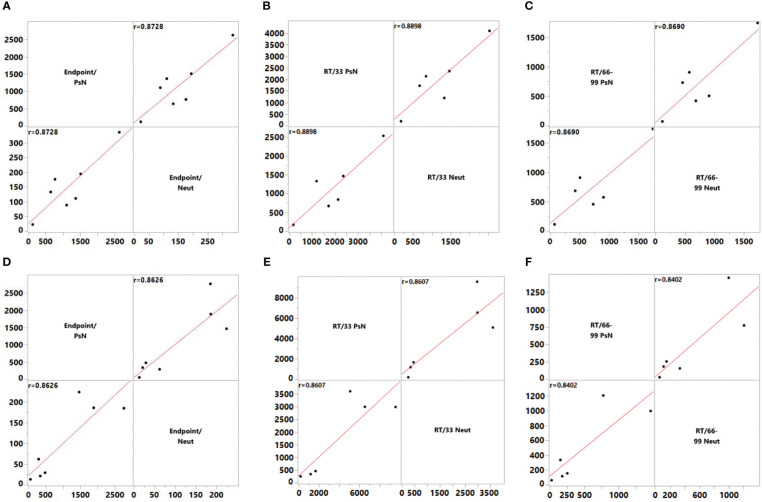
Correlation of endpoint titer and RT/33 and RT/66–99 between the pseudovirus neutralization assay (PsN) and live virus neutralization assay (Neut) methods. **(A)** Endpoint titer, wild-type (WT) strain, **(B)** Titers relative to 33, WT strain, **(C)** Titers relative to 66–99, WT strain, **(D)** Endpoint titer, Delta Variant, **(E)** Titers relative to 33, Delta Variant, **(F)** Titers relative to 66–99, Delta Variant. To assess the level of agreement between the PsN and Neut methods, correlations were estimated using the Row-wise method. Correlation coefficients were calculated using the endpoint titer and the titer relative to samples 33 and 66–99.

First, as shown in **Figure 3**, the endpoint titers, RT/33, and RT/66–99 were used to express the detection results challenged by WT strain. The correlation P values of the Neut and PsN methods were 0.0103, 0.0175, and 0.0246, and the r values were 0.8728, 0.8898, and 0.8690, respectively. When challenged with the Delta variant, the correlation P values of the endpoint titers, RT/33, and RT/66–99 were 0.0125, 0.0278, and 0.0363, respectively, whereas the r values were 0.8626, 0.8607, and 0.8402, respectively. The results expressed by the endpoint titers showed that there was a significant correlation between the results of the two methods (P < 0.05) whether it is the WT strain or Delta variant, and the r values were between 0.86 and 0.87, both of which were well correlated. When samples 33 or 66–99 were used to calibrate the methods, the P value between the two methods was still <0.05, and the r value was between 0.84 and 0.89. This result indicates that the calibration does not change the correlation between the Neut and PsN methods, and that the correlation is still good.

##### 3.3.2.2 GMT difference

To visualize the difference in GMT between the two methods, the GMT Rate of Neut/PsN (Rate _N/P_) for each sample was calculated for WT and Delta strain detection, respectively (**Table 2**). When the WT strain was used, the Rate N/P of the endpoint titer was a 4.3–12.4-fold difference between the Neut and PsN methods. After calibration of samples 33 and 66–99, the Rate _N/P_ decreased to 0.9–2.6- and 0.6–1.6-fold, respectively. When the Delta variant was used, the Rate _N/P_ of the endpoint titer was 4.0–16.7-fold between Neut and PsN method. Calibrated by samples 33 and 66–99, the Rate N/P dropped to 0.7–3.6- and 0.4–1.6-fold, respectively. This indicates that regardless of the WT or Delta variant, the application of samples 33 and 66–99 effectively reduced the variability between the Neut and PsN methods, especially for samples 66–99.

**Table 2:** Endpoint titer, RT/33, RT/66–99 between the Neut and PsN assays.

Challenge Virus	Samples code	Endpoint titer			RT/33			RT/66–99		
		Neut assay	PsN assay	Neut/PsN	Neut assay	PsN assay	Neut/PsN	Neut assay	PsN assay	Neut/PsN
WT strain	11	335	2632	7.8	2530	4105	1.6	1732	1741	1.0
	22	111	1367	12.3	839	2133	2.5	574	905	1.6
	33	133	641	4.8	/	/	/	684	424	0.6
	55	23	117	5.1	172	182	1.1	118	74	0.6
	66–99	194	1512	7.8	1461	2358	1.6	/	/	/
	77	89	1103	12.4	670	1721	2.6	459	730	1.6
	88	176	764	4.3	1326	1192	0.9	908	506	0.6
Delta Variant	11	185	2765	14.9	2980	9573	3.2	997	1464	1.5
	22	29	478	16.7	461	1654	3.6	154	253	1.6
	33	62	289	4.7	/	/	/	335	153	0.5
	55	13	52	4.0	252	180	0.7	60	25	0.4
	66–99	186	1889	10.2	2988	6539	2.2	/	/	/
	77	21	337	15.9	341	1168	3.4	114	179	1.6
	88	224	1467	6.6	3600	5077	1.4	1205	776	0.6

Green represents lower values of RT/33 or RT/66–99 between the Neut and PsN assays than that of the endpoint titer.

To visualize the difference in GMT between the two methods, the GMT Rate of Neut/PsN (Rate N/P) for each sample was calculated for WT and Delta detection, respectively. WT, wild-type; PsN, pseudovirus neutralization assay; Neut, live virus neutralization assay.

When challenged with the WT or Delta variant, samples 66–99 reduced the Rate N/P between the two methods to 0.6–1.6-fold or 0.4–1.6-fold. The results confirmed the consistency and comparability of the test results between the Neut and PsN methods.

### 3.4 Calibration to the WHO IS

#### 3.4.1 Candidate standards

**Figure 4:**
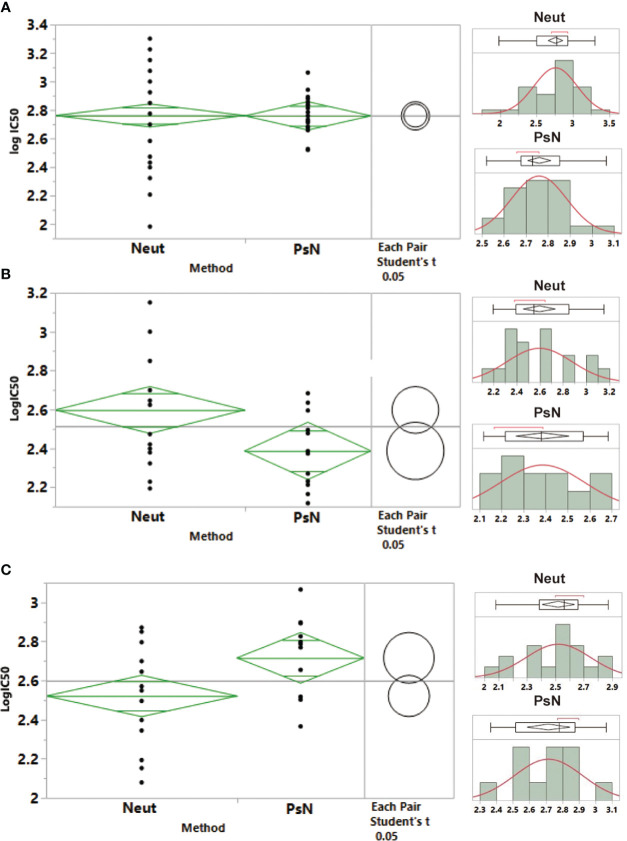
Statistical analysis for the neutralizing antibody potencies of samples 66–99, 33, and 22, and calibration to the international standard. **(A)** Samples 66–99; **(B)** Sample 33; **(C)** Sample 22. A normal distribution analysis of the neutralizing antibody potencies of samples 66–99, 33, and 22 was carried out. One-way analysis of variance was used to analyze the variance between the live virus neutralization assay (Neut) and pseudovirus neutralization assay (PsN).

In this study, the endpoint titers of samples 33 and 66–99 were converted to IU relative to the endpoint titer of the WHO IS. All experimental data were analyzed for normality and homogeneity of variance. The results showed that the IU data of samples 66–99 were all distributed normally using the Neut and PsN methods. There was no significant difference in the geometric mean between the two methods (P=0.9733), but the dispersion degree of the Neut method was greater than that of the PsN method (**Figure 4A**). According to the weighted statistical analysis, the calibrated value of samples 66–99 traceability to WHO IS was 580 (460–740) IU/mL and 580 (520–640) IU/mL in the Neut and PsN methods, respectively. Nevertheless, a significant difference in the geometric mean of sample 33 was found between the two methods (P=0.0314, **Figure 4B**), which led to the assignment not being merged. Therefore, the calibrated values for sample 33 were 400 (320–490) IU/mL and 240 (190–310) IU/mL for the Neut and PsN methods, respectively.

#### 3.4.2 First Chinese NS for SARS-CoV-2 NtAb

As the first NS in China was established before the WHO IS, it was not traceable to the first-generation international standard through collaborative calibration. At the same time, to clarify the quantitative value relationship between the NS and the first generation of NS established, the first NS was also traceable to the WHO IS based on this collaborative calibration. After the sample 22 results were converted to IU by the WHO IS, there was also a significant difference between the two methods (P=0.0239, **Figure 4C**), and the values could not be combined. Therefore, the weighted method was adopted for statistical analysis. The traceability values of sample 22 were 330 (280–390) IU/mL and 520 (410–660) IU/mL for the Neut and PsN methods, respectively.

## 4 Discussion

In December 2020, the WHO Expert Committee on Biological Standardization (ECBS) approved the first WHO International Standard for anti-SARS-CoV-2 immunoglobulin (20/136, 250 IU/ampoule) (22). To harmonize the wide range of methods used globally, a pool of convalescent plasma from 11 COVID-19 patients (NIBSC code 20/136) was evaluated as a candidate IS in a collaborative study. The results showed that 20/136 could effectively demonstrate a decrease in %GCV value among laboratories using different methods, thus addressing the lack of standards for many methods worldwide, and providing a globally unified value for the harmonization and coordination of the detection of COVID-19 antibodies. However, owing to the wide variety of viral targets and classes of immunoglobulin for binding antibodies, an IU could not be assigned for the first WHO IS. The standard was recommended as a comparator; to avoid confusion between the quantification of binding and neutralizing activity, the binding antibody unit (BAU) was introduced (16, 25). At the same time, owing to the extensive demand for IS, the WHO also encourages and supports countries or regions worldwide to develop and apply secondary standards, which not only ensures that the limited IS can be used to maintain the stability and sustainability of quantity traceability but also meets the great demand for vaccines and the related research and development of drugs worldwide. For this purpose, the “WHO manual for the preparation of reference materials for use as secondary standards in antibody testing”, and the “WHO manual for the establishment of national and other secondary standards for antibodies against infectious agents focusing on SARS-CoV2” were issued, which raise the requirements for the development of standards, collaborative calibration, statistical analysis, and assignment, and guide the development and application of national secondary standards systematically globally (26).

As early as September 2020, the Chinese NIFDC established the first generation of China’s national NtAb standard (NS-1st, No. 280034-202001, 1000 U/mL) before the establishment of the WHO IS, which was prepared from Chinese COVID-19 convalescent plasma (19). Collaborative calibration was carried out by 11 laboratories, including the national quality control laboratory, which confirmed that the first generation of NS could effectively reduce the differences in NtAb detection among laboratories, achieving improvements in the accuracy and comparability of NtAb detection among different laboratories and products. The establishment of this standard has played a key role in ensuring the research and development of vaccines and antibodies in China. However, at that time, it could not be traced back to the WHO IS.

For this reason, in 2021, as one of the national quality control laboratories in China and the WHO CC, the NIFDC prepared two candidate standards (lot No. 1 and lot No. 2: CS from COVID-19 convalescent plasma or immunoglobulin, respectively) in accordance with the WHO guidelines and the requirements for the development of reference materials in the Chinese Pharmacopoeia (25–29). This collaborative study included nine laboratories with extensive experience in COVID-19-NtAb detection in China. In total, 10 samples, including three candidate standards (batch number 2 and repeatedly set batch number 1), one first NS, two negative healthy human sera, two WT convalescent sera with different titers, and one Delta convalescent serum, were jointly calibrated to the first WHO IS. The results showed that samples 66–99 could effectively reduce the difference between laboratory tests of all samples (including Delta sera) for the WT strains. The GCV value of the Neut method and the PsN method reduced from 64–148% and 36–115% to 29–88% and 7–54%, respectively. At same time, under the premise of ensuring a good correlation between the Neut and PsN methods (P <0.05, r = 0.8690 for WT; P <0.05, r =0.8402 for Delta), the difference between these two methods could also reduce significantly by standardization with samples 66-99. The ratio for the GMT Neut/PsN method decreased to 0.6–1.6 fold different and 0.4–1.6 fold different from 4.3–12.4 times and 4.0–16.7 times on challenge with the WT strain and Delta variant, respectively. It has been indicated that samples 66–99 can significantly reduce the difference between Neut and PsN methods, and effectively ensure the consistency and comparability of the detection results.

Measurement traceability is the core of reference for material development. To ensure the accuracy and reliability of the measurement value, the WHO specifically documented in the preparation manual of secondary standards and clearly proposed that the calibration value of the secondary standard should include measurement uncertainty (MU), which can be expressed by the 95% confidence limit, and MU should contain requirements for method specificity (27, 28). According to the WHO’s requirements, the test results of samples 66–99 in each test were calibrated to IU/mL according to the test results of the WHO IS in the same test, and the distribution and mean value tests were conducted using the X fitting Y modeling, showing that there was no significant difference (P=0.9733) between the two methods for sample 66–99, but the dispersion degree was different. Following statistical analysis, the values were 580 (460–740) IU/mL and 580 (520–640) IU/mL for the Neut and PsN methods, respectively. However, there was a significant difference between the two methods in terms of the first NS (sample 22) in China (P=0.0239). The assigned values were 330 (280–390) IU/mL and 520 (410–660) IU/mL. The candidate standards and the first-generation NS were traced to the WHO IS with methodically specific research based on statistical analysis at this collaborative calibration, which not only ensures the traceability accuracy and reliability of secondary standards but also clarifies the quantity relationship between new standards and the first NS, ensuring the smooth connection of new standards.

In summary, the secondary standards established by regions or countries are key to ensuring the correct application of WHO IS for practical NtAb detection. Calibration accuracy and property consistency with WHO IS are key to ensuring that the quantity value of the WHO IS is correctly transferred to the working standard, so that the WHO IS can play an accurate role. However, the development of secondary standards is usually ignored and there are few specific research reports on the calibration and traceability of antibodies. According to the “WHO manual for the preparation of reference materials for use as secondary standards in antibody testing” and the “WHO manual for the establishment of national and other secondary standards for antibodies against infectious agents focusing on SARS-CoV2”, the first national standard traceable to the WHO IS, which is the China NS (No. 280034-202102, sample 66-99), was established through collaborative calibration with 580 (460–740) IU/mL and 580 (520–640) IU/mL, respectively, and will be used for the quantitative detection of COVID-19 NtAb (Neut and PsN method). This standard effectively reduced the inter-laboratory detection error of all samples for the WT strain. More importantly, through collaborative calibration research, it was verified that it can significantly reduce the system error between the two methods for the first time without affecting their correlation, suggesting that the application of this standard can effectively ensure comparability and consistency of the detection results between the two methods. As a national quality control laboratory, the NIFDC has also established a robust and reliable COVID-19 NtAb analysis method based on the analytical quality by design (AQbD) and entire life cycle concepts, which can effectively reduce the random error of detection method. The combined application of this method and the secondary standard can achieve the goal of ensuring accurate, comparable, and stable detection of COVID-19 NtAb, and build a scientific foundation for effectively overcoming the challenge of comparing the effectiveness of the COVID-19 vaccine among WHO and regulators around the world. Unfortunately, this standard did not significantly reduce inter-laboratory differences in the Delta strain. Meanwhile, the Omicron and other VOC were not included in this research because of time constraints. In the future, we will pay more attention to the research on the WHO IS, continue research on secondary reference materials for NtAb against Omicron and new emerging VOC variants, and provide sufficient, reliable, and traceable WHO IS reference materials for the research of new vaccines and immune strategies.

## Data availability statement

The original contributions presented in the study are included in the article/**Supplementary Material**. Further inquiries can be directed to the corresponding authors.

## Author contributions

Conceptualization, ZL, MX, and JH; methodology, LG, QM, and DT; formal analysis: JL, XZ, LL, ML, ZW, FC, BC, QH, QW, FG, YW, LB, and XW; writing—original draft preparation, LG and QM; writing, review, and editing, ZL; supervision, DT and MX; funding acquisition, MX. All authors contributed to the article and approved the submitted version.
