# Supplementary material for: Establishment of national standard for anti-SARS-Cov-2 neutralizing antibody in China: The first National Standard calibration traceability to the WHO International Standard
Source: Front Immunol. 2023 Feb 14;14:1107639. doi: 10.3389/fimmu.2023.1107639 (PMC9971588; doi:10.3389/fimmu.2023.1107639)
Supplement: Supplementary file 1 [file DataSheet_1.docx]

Supplementary Material

# Supplementary Figures and Tables

## Supplementary Figures


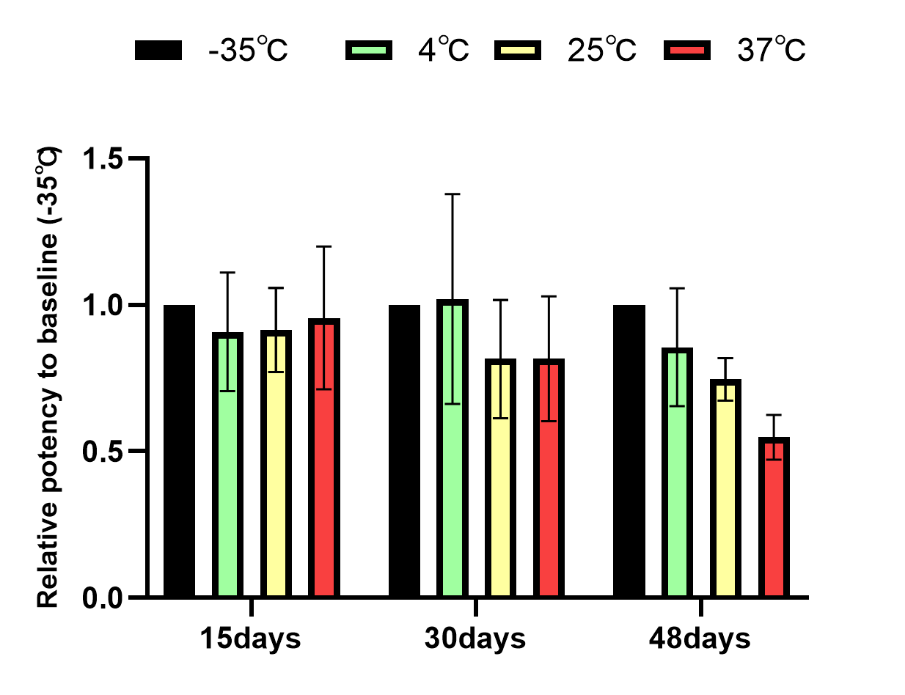


**Supplementary Figure 1.** Thermal degradation assessment of candidate 1. Samples 66/99 were stored at four different temperatures (-35, 4, 25 and 37˚C). At each time point, the potency of our vials were assessed by the pseudovirus neutralization (PsN) assay. Data are reported relative to the baseline storage temperature of -35°C. Error bars represent the 95% confidence limit.

**
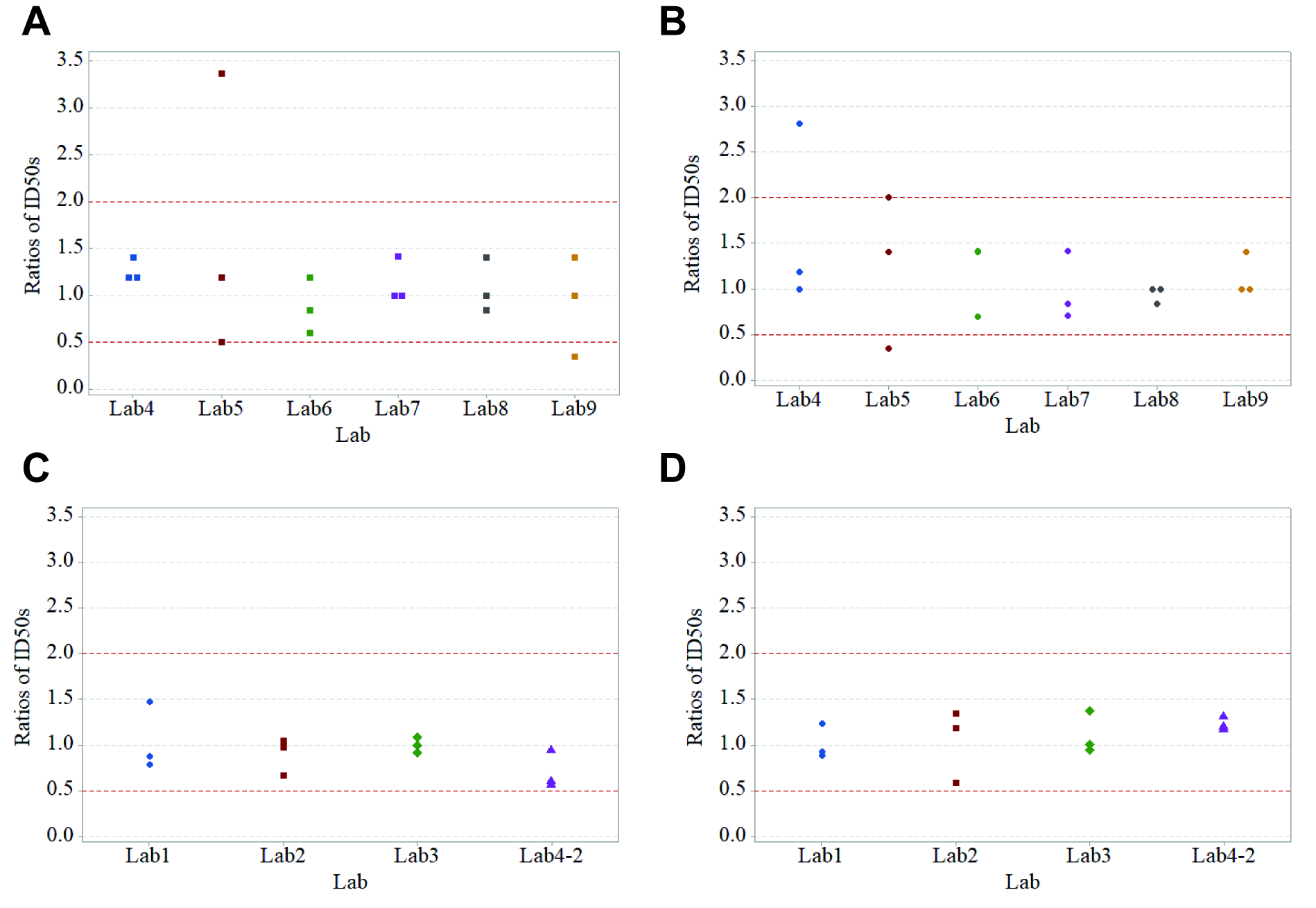
**

**Supplementary Figure 2.** Intra-assay variability in all laboratories. (A) The live virus neutralization assay challenged by wild-type (WT) strain; (B) the live virus neutralization assay challenged by the Delta variant; (C) the pseudovirus neutralization assay challenged by the WT strain; and (D) the pseudovirus neutralization assay challenged by the Delta variant.

Intra-assay variability was analyzed by calculating the ratios of the 50% inhibitory dilutions (ID_50_s) for coded duplicates (66/99) in each assay.

## Supplementary Tables

**Supplementary** **Table 1.** Sample information of the collaborative study

| No. | Sample Code | Samples | Dosage Form |
| --- | --- | --- | --- |
| 1 | 10 | Serum from a SARS-CoV-2-negative healthy human | Liquid |
| 2 | 11 | First WHO International Standard for anti-SARS-CoV-2 immunoglobulin (Coded: 20/136) | Freeze-dried |
| 3 | 22 | First national standard of China for anti-SARS-CoV-2 immunoglobulin (Lot: 280034-202001) | Liquid |
| 4 | 33 | Candidate 2 (Lot: 20200905, the anti-SARS-CoV-2 immunoglobulin) | Freeze-dried |
| 5 | 44 | Serum from a SARS-CoV-2-negative healthy human | Liquid |
| 6 | 55 | Convalescent serum from a donor infected with wild-type SARS-CoV-2 (Low titer) | Liquid |
| 7 | 66 | Candidate 1 (Lot: 202102, a pool of plasma samples from COVID-19 convalescent patients collected before April 2021) | Liquid |
| 8 | 77 | Convalescent serum from a donor infected with wild-type SARS-CoV-2 (High titer) | Liquid |
| 9 | 88 | A pool of sera from COVID-19 recovered patients infected with the Delta variant | Liquid |
| 10 | 99 | A duplicate of sample 66 | Liquid |

**Supplementary Table 2.** Inter-assay variability in all laboratories (Geometric Coefficients of Variation[GCV], %).

| Virus | Samples code | Neut method | | | | | | PsN method | | | |
| --- | --- | --- | --- | --- | --- | --- | --- | --- | --- | --- | --- |
|  |  | Lab4 | Lab5 | Lab6 | Lab7 | Lab8 | Lab9 | Lab1 | Lab2 | Lab3 | Lab4 |
| WT | 10 | / | / | / | / | / | / | / | / | / | / |
|  | 11 | 18.9 | 18.9 | 62.7 | 78.8 | 41.4 | 41.4 | 32.3 | 16.8 | 42.5 | 16.7 |
|  | 22 | 64.9 | 43.4 | 22.2 | 18.9 | 10.5 | **0** | 14.3 | 22.7 | 67.4 | 20.8 |
|  | 33 | 43.4 | 74.5 | **105.7** | 43.4 | 22.2 | 22.2 | 16.4 | 16.9 | 28.8 | 26.5 |
|  | 44 | / | / | / | / | / | / | / | / | / | / |
|  | 55 | 10.5 | 18.9 | 10.5 | 49.2 | **0** | 41.4 | 5.7 | 15 | **78.6** | 13 |
|  | 66 | 35 | 83.8 | 10.5 | 69.8 | 22.2 | 41.4 | 16 | 28.2 | **3.2** | 15.6 |
|  | 77 | 49.2 | 46.7 | 10.5 | 54.7 | 69.8 | 22.2 | 57 | 30.9 | 27.2 | 41.8 |
|  | 88 | 43.4 | 18.9 | 10.5 | 69.8 | 22.2 | 69.8 | 37.5 | 7.2 | 32.2 | 11.6 |
|  | 99 | 30.3 | 41.4 | 30.3 | 41.4 | 18.9 | 49.2 | 45.7 | 27.1 | 15.5 | 34.4 |
| Delta | 10 | / | / | / | / | / | / | / | / | / | / |
|  | 11 | 54.7 | 10.5 | 0 | 69.8 | 22.1 | 49.2 | 27.1 | 44.8 | 15.0 | 59.5 |
|  | 22 | 22.2 | 18.9 | 43.4 | 86.8 | 82.3 | **0** | 18.2 | 17.7 | 59.3 | 42.3 |
|  | 33 | 44.4 | 128.5 | 43.4 | 30.3 | **163.3** | 22.2 | 27.8 | 13.2 | **79.5** | 61.3 |
|  | 44 | / | / | / | / | / | / | / | / | / | / |
|  | 55 | / | 10.5 | / | 64.9 | / | 0 | 30.2 | 28.3 | 67.8 | 12.7 |
|  | 66 | 74.5 | 58.2 | 30.3 | 22.2 | 10.5 | 22.2 | **5.3** | 23.5 | 25.3 | 38.4 |
|  | 77 | / | 69.8 | 30.3 | 41.4 | 18.9 | 0 | 70.0 | 26.4 | 32.2 | 39.5 |
|  | 88 | 11.1 | 10.5 | 10.5 | 68.2 | 10.5 | 22.2 | 19.6 | 6.1 | 32.3 | 26.1 |
|  | 99 | 0 | 58.2 | 30.3 | 18.9 | 18.9 | 22.2 | 14.5 | 27.5 | 22.3 | 27.7 |

/: indicates a laboratory obtained a negative result, and GCV could not be determined.

Inter-assay variability was statistically analyzed as GCVs among the three assays for each assay from all participants. WT, wild-type; PsN, pseudovirus neutralization; Neut, live virus neutralization.
